# Aseptic abscess syndrome: a case report of a patient achieving remission with both infliximab originator and biosimilar administered at varied intervals

**DOI:** 10.3389/fimmu.2024.1454813

**Published:** 2024-12-03

**Authors:** Federica Maria Ucci, Rossana Scrivo, Cristiano Alessandri, Fabrizio Conti, Roberta Priori

**Affiliations:** ^1^ Department of Clinical Internal, Anesthesiologic and Cardiovascular Sciences, Rheumatology Unit, Sapienza University of Rome, Rome, Italy; ^2^ Saint Camillus International University of Health Science, UniCamillus, Rome, Italy

**Keywords:** aseptic abscess syndrome, autoinflammatory disorder, TNFα-inhibitor, biosimilar, non-medical switch

## Abstract

Aseptic abscesses syndrome is a rare but increasingly recognized disease that falls within the spectrum of autoinflammatory disorders. Here, we describe the case of a patient who presented with abdominal pain and fever, along with multiple abdominal and extra-abdominal abscesses, in the absence of underlying hematologic, autoimmune, infectious, or neoplastic conditions. Initially, the patient responded to glucocorticoids, but experienced several flares upon discontinuation, leading to the initiation of treatment with a TNFα inhibitor. After 5 years, an attempt to discontinue treatment resulted in a new flare of the disease. Remission was eventually achieved with a biosimilar TNFα inhibitor, albeit requiring shortened infusion intervals.

## Highlights

- Aseptic abscesses syndrome (AAS) may extend to clinically silent sites, suggesting that a widespread imaging study may be useful to define the involvement of the disease.
- In patients with AAS treated with bDMARDs and in persistent remission, spacing can be considered, while the withdrawal of treatment may expose the patients to a risk of disease flare.
- In patients with AAS the non-medical switch to a biosimilar TNFα inhibitor may yield a similar efficacy outcome with respect to the biooriginator.

## 1 Introduction

Aseptic abscesses syndrome (AAS), first described in 1995 (1), is a rare yet emerging disease categorized within the expanding spectrum of autoinflammatory disorders (2). The latest literature review includes 71 patients from the French AAS register. The abscesses were primarily located in the spleen (71.8%) and lymph nodes (50.7%), but were also found in the skin, liver, and lung. Rarer locations include the brain, genitals, kidneys, ENT, muscles, and breasts. Approximately 48% of patients presented with multiple abscesses, with an average of 1.3 ± 1.6 organs affected at diagnosis, increasing to 2.5 ± 1.5 over the disease course. Most patients had an associated condition, primarily IBD. Among these patients, 61.9% experienced a relapse, typically within 1 year of diagnosis, with hepatic and skin abscesses at diagnosis being the main associated risk factor (3). Aseptic abscesses are primarily lesions in internal organs, particularly in the abdominal region, accompanied by local and systemic clinical or biological manifestations. At the anatomopathological level, they resemble those observed in Sweet syndrome, except that their topography is dermoepidermal. Only 20% of patients with aseptic abscesses also present with associated neutrophilic dermatosis, and the reason these profound manifestations develop on different skin lesions remains unknown. The pathophysiology of ASS is largely unclear. However, the presence of associated conditions suggests several hypotheses. Similar to pyoderma gangrenosum and Behçet’s disease, vasculitic phenomena could play a role, as vessels filled with polymorphonuclear neutrophils (PNN) are sometimes observed at the periphery of aseptic abscess lesions, but this may merely reflect the influx of PNN into the inflammatory site before their migrate toward the abscess. In addition, pathological studies of aseptic abscesses consistently show an absence of vasculitis. Although there is no strong indication of autoimmunity, immune complex-mediated vasculitis mechanisms cannot be completely ruled out. The strong association between aseptic abscesses and IBD suggests the presence of shared pathophysiological mechanisms. A defining characteristic of aseptic abscesses is their neutrophilic component. Neutrophils are key cells of innate immunity and are implicated in both monogenic and polygenic autoinflammatory diseases such as Crohn’s disease. In IBD, which is considered a disease of barrier organs, there is increased intestinal permeability. This barrier disruption could facilitate the dissemination of non-viable microbial particles or lead to aberrant “homing” of memory T lymphocytes/intestine-associated effectors or PNN via altered integrins, resulting in distant inflammatory lesions (4, 5). PAPA syndrome is also part of this family of diseases, and the efficacy of anakinra suggests an important role of the inflammasome in its pathogenesis [49]. Therefore, AAS can be compared to the group of autoinflammatory diseases. However, while an association between aseptic abscesses and the CD2BP1/PSTPIP1 gene, implicated in PAPA syndrome, has been investigated, no such association has been found (6). The syndrome typically manifests in young adults with fever, leukocytosis, elevated erythrocyte sedimentation rate (ESR) and C-reactive protein (CPR), and abdominal pain, alongside radiological evidence of aseptic abscesses (7). Currently, diagnostic criteria and management guidelines are lacking, with the literature primarily comprising case reports and case series. Therefore, ASS is a diagnosis of exclusion, made by ruling out infectious abscesses, including mycobacterial infection, neoplasms (particularly lymphoma), chronic septic granulomatosis, and inflammatory and autoimmune conditions (such as sarcoidosis, granulomatosis with polyangiitis, or rheumatoid nodules). While antibiotics show no efficacy, remission is generally achieved with GCs which are widely used as the first-line treatment to induce remission. However, despite a positive response to GCs in approximately 95% of patients (with initial dosages ranging from 0.5-1 mg/Kg/day and tapering starting from the second to fourth week), such treatment is associated with a high relapse rate (1, 2, 7, 8), along with known adverse effects. Conventional synthetic disease modifying antirheumatic drugs (csDMARDs) and/or biologic DMARDs (bDMARDs) may be required as steroid-sparing agents and to maintain remission (8–10). CsDMARDs used to treat AAS include azathioprine, colchicine, cyclophosphamide, ciclosporin, methotrexate, and mycophenolate mofetil. Data on the efficacy of bDMARDs remain limited, with only a few case reports involving the use of TNF antagonists, anti-IL1ß, and anti-IL6R agonists (9, 10). According to the literature, complete response has been achieved in most cases treated with biologics, showing rapid improvement (20 out of 26 patients) (3). Although data remain scarce, bDMARDs, particularly TNF antagonists, appear effective in managing AAS. Generally, the prognosis is favorable; however, it may be influenced by the presence of an underlying condition. Due to its rarity, even a single documented case can be noteworthy. The case described is the first reported attempt at discontinuing bDMARDs, followed by the first non-medical switch of the TNF antagonist infliximab.

## 2 Case report

We report the case of a 51-year-old woman with an unremarkable medical history until November 2010, when she was 37 years old. At that time, she began experiencing abdominal pain and fever (up to 40°C) preceded by shivering, without any apparent triggering causes, while her bowel function remained normal. A CT scan of her abdomen revealed multiple liver abscesses and several mesenteric lymphadenopathies, including conglobate ones. A diagnosis of necrotizing granulomatous hepatitis with abscess formation was established. Despite negative results from serological tests, blood, fecal, and urine cultures, as well as liver biopsy with PAS and GIEMSA staining, empiric antibiotic therapy (levofloxacin and penicillin), along with antiparasitic medication (mebendazole) and GCs was initiated, resulting in resolution of fever. In November 2011, several months later, she was readmitted to the hospital due to a recurrence of the same clinical presentation upon withdrawal of GCs. CRP levels were elevated (45 mg/L), along with an increased platelet count (PLT 773 x 109/L) and neutrophil count (WBC 14.5 x 109/L, N 58.8%). Infectious diseases were reasonably ruled out (negative results for anti-CMV, anti-Toxoplasma, anti-Treponema, anti-Echinococcus, QuantiFERON TB-Gold test, VDRL, anti-EBV, blood, fecal and urine cultures), as were autoimmune disorders (negative results for c-ANCA, p-ANCA, AMA, ASMA, ANA, antibodies against LKM-1, Ro-52, gp210, sp100, and SLA/LP).

An abdominal MRI revealed multiple focal lesions within the spleen and liver, some of which were partially confluent with a necrotic central core (images not available). Pathological analysis of liver abscess biopsy showed a pronounced inflammatory infiltrate predominantly composed of neutrophil granulocytes with a significant presence of eosinophils. Following an initial remission with GCs, a new flare of symptoms and laboratory abnormalities recurred three months after discontinuation. Despite high-dose intravenous GCs, there was no response, prompting the initiation of intravenous Cyclophosphamide at 750 mg every 21 days (for a total of 11 infusions), resulting in rapid resolution of fever and gradual clinical improvement. Azathioprine at a dose of 100 mg per day was subsequently started for maintenance therapy.

**Figure 1:**
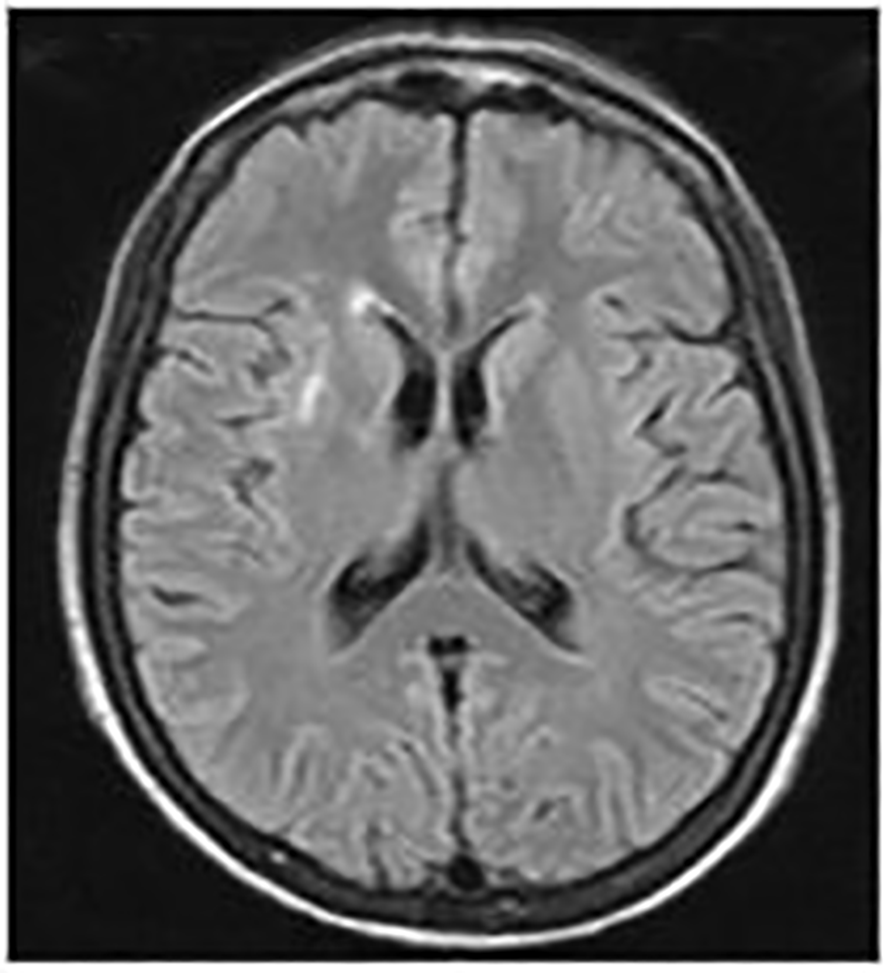
Brain MRI scans. September 2012: A hyperintense area on T2 and FLAIR sequences was observed in the anterior arm of the right external capsule, with a maximum diameter of 21 mm. The lesion exhibited an elongated morphology and post-contrast enhancement suggestive of barrier disruption near vascular structures, consistent with the patient's inflammatory pathology.

Over the following years, despite undergoing treatment, the patient experienced recurring episodes of high fever. In 2013, MRI scans revealed a consistent enlargement of splenic abscesses, prompting the decision to perform a splenectomy and tangential resection of the gastric fundus. Concurrently, a brain MRI was conducted, showing a lesion consistent with the patient’s inflammatory pathology (**Figure 1**).

In June 2014 the patient experienced a new flare of the disease characterized by fever (with a temperature of 40°C) accompanied by a nodular skin lesion on the left leg.

An abdominal MRI (images not available) revealed a general increase in the number of abscess lesions, affecting the lung parenchyma, the peri-pancreatic area, the liver, and the left paraaortic area.

In September 2014, four years after the onset of her symptoms, the patient came to our attention. Laboratory examinations revealed anemia (Hb 8.3 g/dL), leukocytosis (WBC 24.57 x 109/L, Neutrophils 19.34 x 109/L), and an increased platelet count (PLT 1.392 x 109/L). There were no signs or symptoms of inflammatory bowel disease (IBD). Once again, autoimmune, infectious, and neoplastic disorders were reasonably excluded. In particular, autoantibody testing (including ANCA, AMA, ASMA, and ANA) was repeated, with all results returning negative. A Quantiferon test was also performed, and a swab of a skin abscess on the left leg was taken (remaining sterile after 72 hours), ruling out autoimmune and infectious disorders. Skin biopsy revealed an abscessed, inflammatory, giant-cellular granulomatous infiltrate with numerous eosinophils. Additionally, ophthalmologic and hematologic consultations showed no evidence of vasculitic or neoplastic lesions. After excluding both infectious and inflammatory/autoimmune and neoplastic diseases and considering the patient’s medical history, it was possible to make the diagnosis of AAS.

Considering the patient’s medical history and the strong performance and substantial data supporting TNF antagonists as steroid-sparing agents in case reports, treatment was initiated with three boluses of high-dose intravenous methylprednisolone, followed by oral tapering. Infliximab (Remicade) was then introduced at a dosage of 5 mg/kg, administered intravenously at T0, followed by subsequent infusions of 5 mg/kg at weeks 2 and 6, and then every 8 weeks, following protocols outlined in previous case reports. Azathioprine dosage was reduced to 50 mg/day, resulting in progressive clinical, laboratory, and instrumental improvement, which persisted even after the discontinuation of GCs.

**Figure 2:**
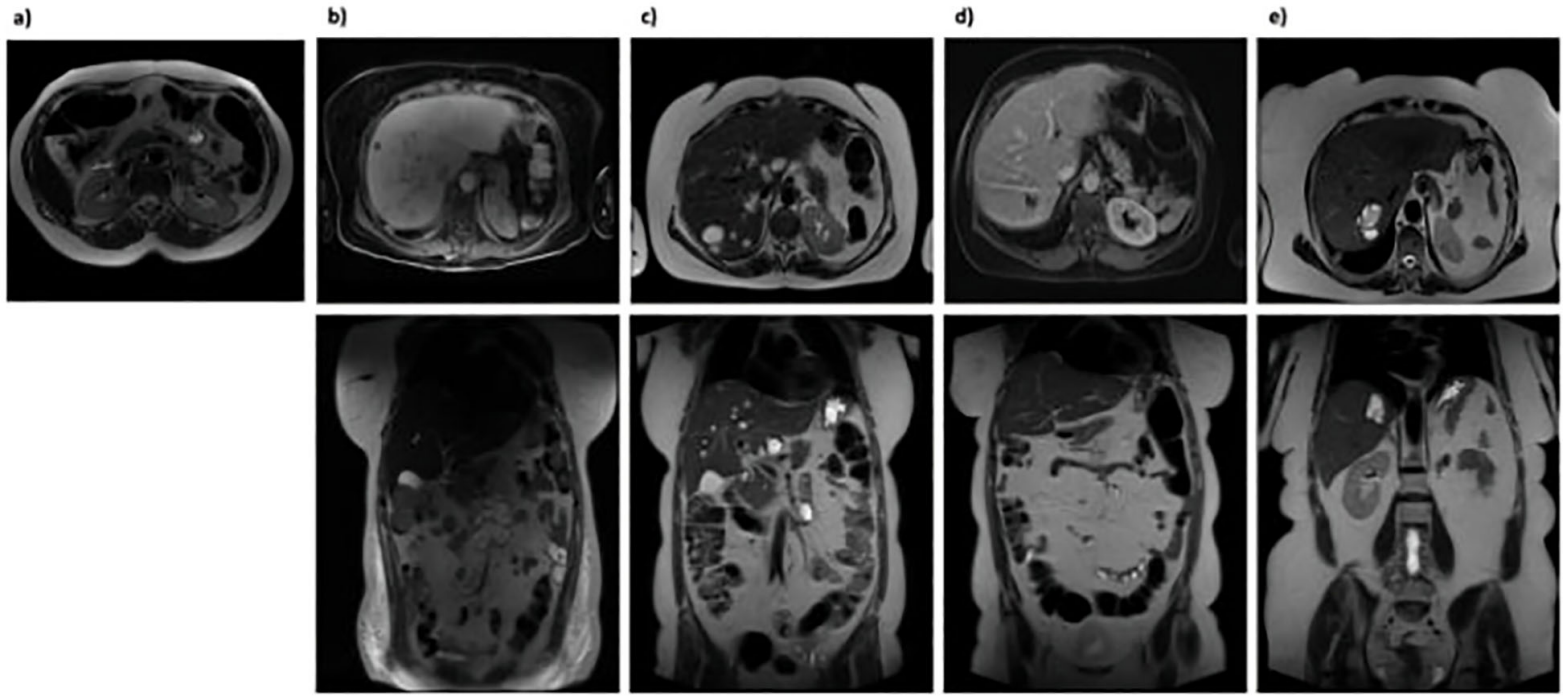
Abdominal MRI scans. **(A)** December 2017: No evidence of abdominal abscess formation; **(B)** July 2020: Multiple hyperintense hepatic lesions appeared across all segments, not previously seen, suggesting potential abscess sites. A large, multiloculated fluid-corpuscular collection measuring 5.5 x 6.5 x 7.5 cm was also noted at the mesenteric root; **(C)** January 2021: Increase in both size and number of multiple nodular formations consistent with abscesses in the intrahepatic region; **(D)** September 2021: Overall reduction in size and number of the intrahepatic nodular formations. The previously identified multiloculated collection at the mesenteric root was no longer discernible; **(E)** April 2024: Marked reduction in intrahepatic nodular formations.

The patient had experienced good health for several years, achieving complete remission, defined by the absence of clinical symptoms (such as fever, abdominal pain, and cutaneous abscesses), normal blood tests (including CRP, ESR, leukocyte count), and the absence of abscesses on MRI follow-up (**Figure 2A**).

After five years, in November 2019, due to sustained clinical and laboratory improvement, which had permitted a gradual spacing of the intervals between Remicade administrations to 16 weeks, treatment was discontinued with the patient’s consent.

However, after six months, the patient experienced a severe relapse characterized by fever (with a body temperature reaching 40°C) and the development of a firm, elastic swelling on the left pretibial area. She was admitted to our hospital, where blood tests revealed leukocytosis (WBC 24.89 x 109/L, Neutrophils 19.91 x 109/L), thrombocytosis (PLT 1.145 x 109/L), elevated CRP (16.01 mg/dL) and severe anaemia (Hb 6.4 g/dL). Infectious disease screening yielded negative results. However, abdominal MRI showed multiple abscess formations in the liver and a substantial collection at the root of the mesentery (**Figure 2B**). The patient was discharged with a diagnosis of exacerbation of the underlying disease and an indication to resume therapy with oral GCs (starting at 37.5 mg/day) and biosimilar infliximab (Flixabi) at a dosage of 5 mg/kg every 8 weeks.

**Figure 3:**
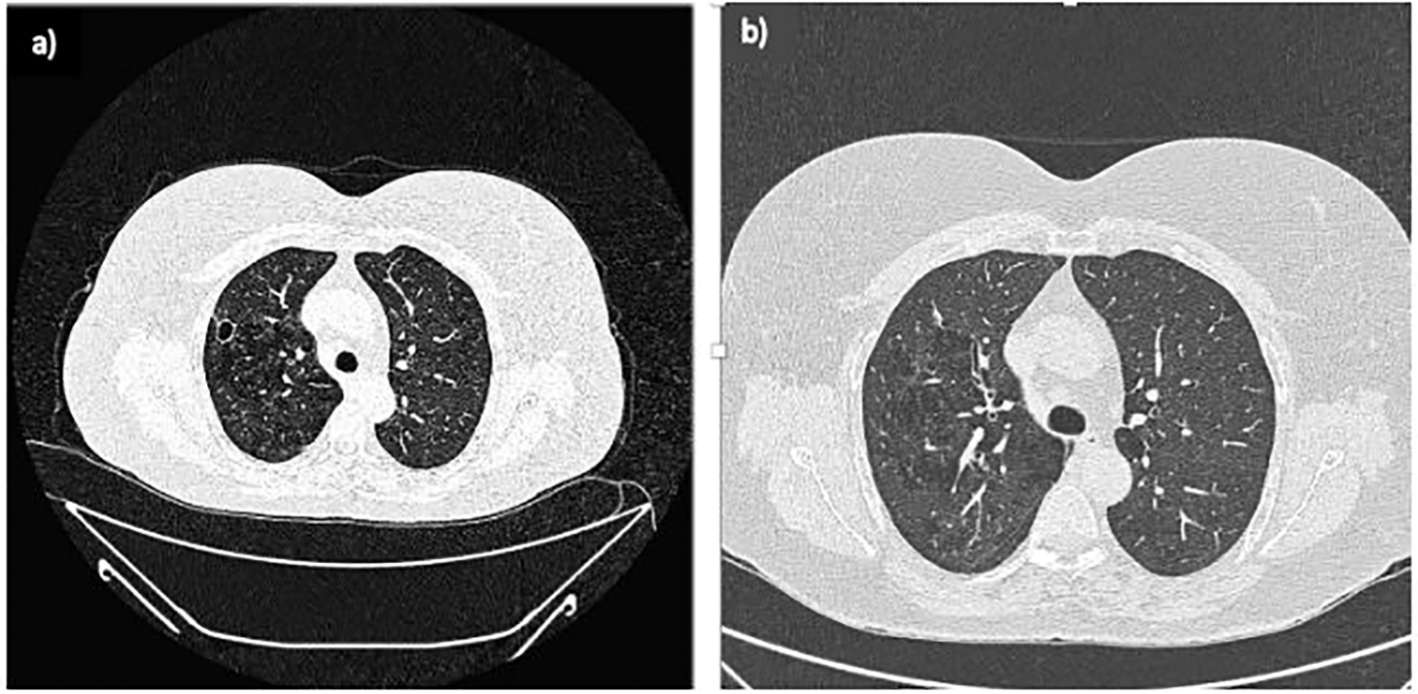
Lung CT scans. **(A)** February 2021: A newly observed nodular formation with a large, air-filled central cavity and thick walls, showing connections to the parietal pleura. This finding suggests a cavitated lesion resulting from an inflammatory process in the resolution phase, consistent with the patient's medical history; **(B)** September 2021: The previously noted cavitated lesion in the right upper lobe shows signs of fibrotic scarring, with the air component no longer visible.

Despite the initial improvement, in December 2020, the patient experienced fever and abdominal pain. Blood tests revealed leukocytosis (WBC 24.17 x 109/L, Neutrophils 15.88 x 109/L), thrombocytosis (PLT 896 x 109/L), anaemia (Hb 10.1 g/dL), and elevated inflammation markers (CRP 282.8 mg/L, ESR 108 mm/h). An abdominal MRI showed a reduction in the volume of abscesses at the mesentery root but an increase in both the number and volume of hepatic abscesses (**Figure 2C**). A chest CT with contrast revealed a cavitated lesion in the right upper lobe consistent with the underlying disease (**Figure 3A**). Subsequently, bronchoalveolar lavage was performed to rule out infectious or neoplastic pathologies, yielding negative results. Therefore, the lung cavitation was ascribed to the underlying disease and treatment with ev GCs was started.

Intravenous methylprednisolone (125 mg) was then administered over three consecutive days, and the interval between infliximab infusions was reduced to 6 weeks, resulting in progressive improvement. This was evidenced by the reduction in inflammation markers (CRP 9.42 mg/L, ESR 40 mm/h) and leukocytosis (WBC 16.6 x 109/L, Neutrophils 7.87 x 109/L), as well as the resolution of thrombocytosis and anaemia (PLT 314 x 109/L, Hb 15.3 g/dL). Over the following months, the patient reported clinical well-being.

**Figure 4:**
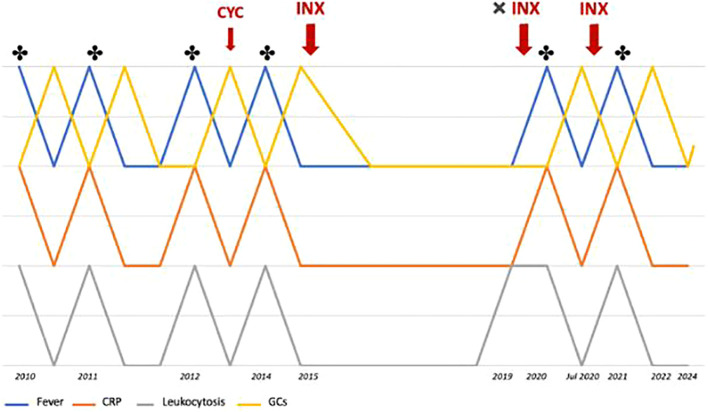
Timeline showing the trends of fever, CRP levels, leukocyte count, and glucocorticoid therapy throughout the patient's clinical history. * indicates the detection of abscesses (MRI or CT); CYC, cyclophosphamide; INX, infliximab.

In September 2021, a follow-up MRI revealed a significant reduction in both the size and number of hepatic abscesses, along with the resolution of the multiloculated fluid-corpuscular collection located at the root of the mesentery (**Figure 2D**). Additionally, a chest CT demonstrated fibrotic scarring evolution of the previously described cavitated lesion in the right upper lobe (**Figure 3B**). Over the past 2 years, the patient has consistently reported clinical well-being and has undergone annual abdominal MRI scans, which have shown a marked reduction in nodular formations in the liver and no other lesions (**Figure 2E**). **Figure 4** displays the trends of clinical, laboratory, and therapeutic parameters of the patient.

## 3 Discussion

AAS is a rare condition, with only a recent extensive description of 71 patients (3). In this report, we present the first documented case of isolated AAS in Italy. Our patient exhibited all the features previously outlined by Andre and colleagues: the presence of deep abscesses, negative screening for infectious diseases, failure of broad-spectrum antibiotic therapy, rapid clinical improvement upon GC therapy, followed by radiological evidence of abscess resolution (1). Furthermore, as previously mentioned, the described cases usually present from one up to three affected organs, while our patient presented a rare involvement of several (up to five) organs, including rare extra abdominal sites, such as brain and lungs. This could be the mirror of a particular aggressive disease in literature panorama, which could also partially explain the starting difficulties in disease management and the need in bDMARDs to obtain a low disease activity.

No other overlapping conditions were demonstrated in this patient; however, we cannot exclude the possibility that the development of another inflammatory condition may have been prevented by the prolonged immunosuppressive and anti-cytokine treatment. In fact, AAS occasionally precedes the onset of Crohn’s disease (1) or is associated with a wide spectrum of disorders such as IBD, Behçet’s disease, SAPHO syndrome, Cogan syndrome, rheumatoid arthritis, and myelodysplastic syndrome (8, 11–16). Due to the lack of diagnostic criteria, the diagnosis was made following an extensive process of excluding other potential causes.

The absence of management guidelines has made it challenging to make timely therapeutic decisions. Data regarding the role of splenectomy are highly controversial: in one case, it resulted in prolonged remission (17), while in another case, the patient developed multiple cutaneous abscesses two weeks after the procedure (18). In our experience, splenectomy did not contribute to achieving remission, aligning with the recommendation against early use of this procedure once the diagnosis has been established.

The optimal management strategy is still evolving: csDMARDs and bDMARDs have been used as maintenance therapy with varying degrees of success. The most frequently reported approach involves the use of TNFα inhibitors, although treatments with anti-IL1 and anti-IL6R agents have also been described, showing promising clinical responses, particularly in patients who have experienced a flare during TNFα inhibitor treatment (3, 9, 10, 19, 20).

In particular, Weins et al. suggest that individual cytokine profiling could contribute to understanding the underlying mechanisms driving inflammation and pave the way for personalized targeted therapy (20). In the present case, based on the most consistent data found in the literature, we opted for a TNFα inhibitor, infliximab (9, 20, 21), which enabled the withdrawal of GCs, leading to stable remission when combined with azathioprine.

To the best of our knowledge, we present the first case in which an initial attempt to extend the interval between infliximab infusions was successfully undertaken. However, complete cessation of the drug resulted in a severe relapse of AAS. Furthermore, we described the first case of non-medical switch to the biosimilar infliximab: based on our observations, the non-medical switch yielded a similar efficacy profile, although it was necessary to shorten the infusion intervals.

However, it is not possible to determine whether this was due to the lower efficacy of the biosimilar or to very high disease activity. A more comprehensive understanding of the natural course of AAS, along with the development of specific diagnostic criteria and management guidelines, is essential to facilitate earlier recognition and improved disease management. There is still an unmet need in this regard. A multicenter prospective study is recommended to further enhance our understanding of this rare condition.

## Data Availability

The original contributions presented in the study are included in the article/supplementary material. Further inquiries can be directed to the corresponding author.
